# Antioxidant Activity of Resveratrol Diastereomeric Forms Assayed in Fluorescent-Engineered Human Keratinocytes

**DOI:** 10.3390/antiox11020196

**Published:** 2022-01-20

**Authors:** Ilaria Bononi, Paola Tedeschi, Vanessa Mantovani, Annalisa Maietti, Elisa Mazzoni, Cecilia Pancaldi, Vincenzo Brandolini, Mauro Tognon

**Affiliations:** 1 Department of Translational Medicine and for Romagna, University of Ferrara, 44121 Ferrara, Italy; bnnlri@unife.it; 2 Department of Chemical, Pharmaceutical and Agricultural Sciences-DOCPAS, University of Ferrara, 44121 Ferrara, Italy; paola.tedeschi@unife.it (P.T.); annalisa.maietti@unife.it (A.M.); elisa.mazzoni@unife.it (E.M.); vincenzo.brandolini@unife.it (V.B.); 3 Laboratories of Cell Biology and Molecular Genetics, Section of Experimental Medicine, Department of Medical Sciences, School of Medicine, University of Ferrara, 44121 Ferrara, Italy; vanessa.mantovani@unife.it (V.M.); cecilia.pancaldi@unife.it (C.P.)

**Keywords:** oxidative stress, resveratrol, diastereomeric mixture, keratinocyte

## Abstract

Resveratrol is a powerful antioxidant molecule. In the human diet, its most important source is in *Vitis vinifera* grape peel and leaves. Resveratrol exists in two isoforms, cis- and trans. The diastereomeric forms of many drugs have been reported as affecting their activity. The aim of this study was to set up a cellular model to investigate how far resveratrol could counteract cytotoxicity in an oxidant agent. For this purpose, a keratinocyte cell line, which was genetically engineered with jelly fish green fluorescent protein, was treated with the free radical promoter Cumene hydroperoxide. The antioxidant activity of the trans-resveratrol and its diastereomeric mixture was evaluated indirectly in these treated fluorescent-engineered keratinocytes by analyzing the cell number and cell proliferation index. Our results demonstrate that cells, which were pre-incubated with resveratrol, reverted the oxidative damage progression induced by this free radical agent. In conclusion, fluorescent-engineered human keratinocytes represent a rapid and low-cost cellular model to determine cell numbers by studying emitted fluorescence. Comparative studies carried out with fluorescent keratinocytes indicate that trans-resveratrol is more efficient than diastereomeric mixtures in protecting cells from the oxidative stress.

## 1. Introduction

Oxidative stress is defined as a set of alterations at molecular, cellular, and tissue levels in a living organism. In the event of excessive exposure to oxidizing agents, modification in the redox balance inside cells arises between the reactive oxygen species (ROS) and the antioxidant system, which favors ROS [1].

ROS is constantly produced in cells through a variety of biochemical processes [2]. At moderate concentrations, ROS are actively involved in complex biological processes, such as control of gene expression, apoptosis, and signal transduction [3]. However, at a high concentration, ROS cause cell damage, such as aging and alterations in cellular constituents [4]. Eukaryotic cells have developed an antioxidant system including endogenous compounds, which work synergistically with exogenous molecules to neutralize free radicals [5] such as ROS. Antioxidants are molecules that are capable of protecting cells against the oxidation induced by free radicals, by blocking the initiation phase of radical production or neutralizing radicals formed during the propagation phase [5].

**Figure 1:**
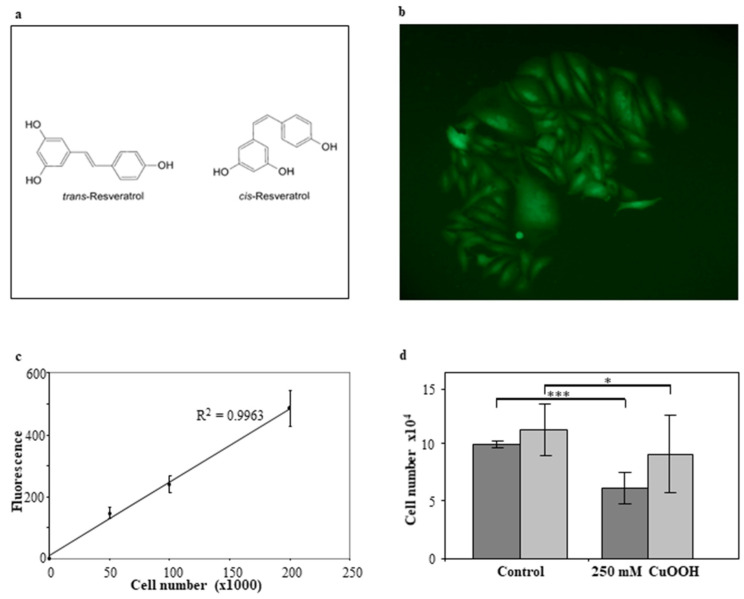
Resveratrol structures, florescent human keratinocyte cells, relation between the number of NCTC-GFP cells and fluorescence intensity, and the effect of CuOOH treatment: Panel (**a**): Resveratrol cis- and trans-isomers chemical structures. Panel (**b**): Microphotograph showing fluorescent NCTC-GFP (magnitude 20X). Keratinocytes exhibit small differences in morphology/shape due to the micro-environment in which they are expanding, i.e., cells inside the monolayer are more rounded, whereas cells growing at the edge of the monolayer are more elongated. Panel (**c**): Relation between the number of NCTC-GFP cells and fluorescence intensity. Samples containing an increased number of cells, ranging from 3.125 × 10^3^ to 10^5^ cells/cm^2^, were analyzed by spectrofluorimetric reading. Five different sets of samples were tested at different times. Each value was reported in the graph as a function of the corresponding cell number. Bars represent the standard deviation. The linear relation between cell number and fluorescence intensity was represented by the R^2^ value (0.9963). Panel (**d**): Effect of CuOOH treatment on cell number (dark gray) and proliferation (light gray). Horizontal bars indicate a statistically significant difference (*: *p* < 0.05; ***: *p* < 0.0005).

Resveratrol is one of the most highly investigated antioxidant molecules (Figure 1a) [6]. Since resveratrol is also a phytoalexin, its biosynthesis starts from the plant’s secondary metabolism, which is stimulated by microorganism infection or abiotic elicitors [7]. While flavonoids are found throughout the plant kingdom, different resveratrol forms have been isolated from only a few plant families. The most important sources of resveratrol in the human diet are grapes, peanuts, berries, and their byproducts. In plants and their derived foods, resveratrol exists in various forms, such as aglycone, glucoside (piceid), and several oligomers. A mixture of cis and trans isomers was identified for each form [8].

The most important source of resveratrol in the human diet is *Vitis vinifera* grape peel and leaves [9,10], which are used for the wine production. In *Vitis vinifera*, 16 resveratrol forms have been detected in grape stems, seeds, skins, and juice. Stems contain the richest source of stilbenes, followed by grape skins. These byproducts of the winemaking processes could provide useful information for determining how to enrich potential health-promoting compounds [11]. Resveratrol contains chemicals that determine its ability to inhibit the progress of certain infections. In general, stressful factors induce an accumulation of resveratrol in plants. This mechanism allows plants to resist parasites and other adverse conditions. Indeed, resveratrol is produced by more than 70 different species of plants in response to such types of stressful situations [12,13]. Given its wide variety of pharmacological properties, resveratrol was indicated as a probable explanation for the so called “French paradox”. Indeed, some epidemiological studies revealed an inverse correlation between wine consumption and cardiovascular diseases in France, a country known for its high intake of saturated fatty acids [13,14] and wine. Resveratrol demonstrates several biological activities such as anti-inflammatory, vasorelaxing, anticancer, antiaging, anti-frailty, as well as antiallergenic characteristics [13,15]. However, the key relevance of resveratrol is due to its antioxidant activity and its behavior as an important radical scavenger [10].

Published studies indicate that resveratrol has a powerful antioxidant activity associated with the presence of three hydroxyl groups in its structure. Resveratrol has an inhibitory effect on excessive ROS production, aberrant mitochondrial distribution, and lipid peroxidation [16,17,18]. Resveratrol leads to an increase in endogenously generated glutathione, and a quantitative reduction in the cellular redox environment and endogenous ROS production [19]. In vitro studies have demonstrated that resveratrol shows a role in maintaining cellular redox homeostasis by preventing an increase in ROS production and a decrease in mitochondrial membrane potential [20]. Resveratrol decreases mitochondria fragmentation and maintains the potential of the mitochondrial membrane while preventing oxidative phosphorylation attenuation, thus exerting a protective effect against the harmful impact of ROS [16]. Resveratrol treatment protects cells from the oxidative damage induced by hydrogen peroxide. This protection is caused by reduced malondialdehyde (MDA) and intracellular ROS concentrations and increased expression levels of antioxidant enzymes [21]. In vivo studies on patients affected by diabetes demonstrate that the protective effects of chronic resveratrol administration is beneficial in: reducing advanced glycation end product (AGE)-induced oxidative stress and apoptosis [22]; normalizing antioxidant status, exacerbated by oxidative stress induced by hyperglycemia [23]; reducing ROS production, elevating membrane potential, and inhibiting cytochrome c release from the inner mitochondrial membrane [24]; and contrasting the 3-nitrotyrosine accumulation and 4-hydroxynonenal generation, increasing total antioxidant capacity [25]. Resveratrol protects the spinal cord from ischemic damage in rats by reducing plasma levels in nitrite, advanced oxidation protein products (AOPP) and MDA, and increasing the enzymatic activity of superoxide dismutase (SOD) and catalase (CAT) [26]. In vivo studies demonstrate that resveratrol also attenuates (i) oxidative stress in rats with experimental periodontitis, (ii) early Alzheimer’s disease, and (iii) chronic obstructive pulmonary disease [27,28,29].

The above mentioned in vitro and in vivo studies indicate that resveratrol has a therapeutic effect in cells and animals suffering from increased oxidative stress, which is associated with a limitation in ROS generation and stimulation of compounds that act as an antioxidant barrier [16].

Resveratrol is characterized by two phenolic rings joined by a styrene double bond, with hydroxyls in position 3 and 5 of the first ring and 4 of the second ring [30]. Resveratrol is a low molecular weight molecule of 228 Dalton [31]. It exists in two diastereomeric forms, cis- and trans-, and its isomerization from the trans- to cis-form is favored by exposure to UV radiation [32]. A diastereomeric mixture is also known as a diastereomers blend, where the proportions of diastereomers may affect its biological activity, and its properties can significantly differ from those of its two individual diastereomers [33,34].

In order to study the effect of the isomer forms of resveratrol, a new in vitro cellular model was set up, represented by fluorescent-engineered human keratinocytes. These cells were employed for measuring in vitro viability. Indeed, cell viability may be used as an indirect parameter of oxidative damage. The fluorescent-engineered human keratinocytes developed in this investigation may represent an alternative study model, which is able to complement animal experimentations.

In our experimental model, Cumene hydroperoxide (CuOOH) was used as a free radical promoter [35]. As reported above, resveratrol as a radical scavenger is likely to contrast the effect of the CuOOH neutralizing radicals that arise from its action.

The aim of this study is to test different forms of resveratrol for their potential antioxidant activity in fluorescent-engineered human keratinocyte cells for the first time. This innovative approach allowed us to investigate whether a diastereomeric mixture shows quantitative differences in antioxidant activity compared to trans-resveratrol.

## 2. Materials and Methods

### 2.1. Cells and Medium

NCTC-2544 human keratinocyte cells (ICN Flow, Irvine, UK) were cultured in a 1:1 mixture of DMEM medium and Ham’s F-12 with Hepes, L-glutamine, 10% FBS, and penicillin/streptomycin (Lonza, Basel, Switzerland).

### 2.2. Fluorescent-Engineered Human Keratinocytes

In order to determine adequate concentrations of the antibiotic Geneticin (G418) in human keratinocytes, NCTC-2544, cells were plated at a density of 5 × 10^4^ cells/cm^2^ with different concentrations of G418, ranging from 0 to 1200 µg/mL. The proportion of viable cells was assessed by trypan-blue staining. Subsequently, NCTC-2544 human keratinocytes parental cells were transfected with the recombinant vector named pIRES2-AcGFP1, using the FuGENE HD Transfection Reagent (Roche, Milan, Italy). The pIRES2-AcGFP1 vector contains coding sequences for the green fluorescent protein from Aequorea coerulescens (AcGFP). After 15–20 days of antibiotic treatment, resistant clones were isolated with sterile glass cylinders and expanded as independent cell lines.

### 2.3. Immunofluorescence Technique

In order to undertake immunofluorescence assays, cells were cultured on coverslips for 18 h, and fixed with 4% paraformaldehyde (PFA). Subsequently, cells were permeabilized and incubated for 1 h at 37 °C with TRITC-conjugated Phalloidin. (Sigma-Aldrich, Milan, Italy), diluted 0.2 μg/mL in PBS 1×. After applying the DAPI solution, the glass coverslips were mounted with glycerol/PBS 9:1. Images were obtained using a TE 2000-E fluorescent microscope. Digital images were captured using ACT-1 and ACT-2 software for DXM1200F digital cameras (Nikon Instruments, Sesto Fiorentino, Italy).

### 2.4. Cell Quantification

NCTC_2544_AcGFP cells were seeded in 24-well plates at different concentrations, ranging from 2.5 × 10^4^ to 1 × 10^5^, which were then used to evaluate the cell number by measuring the cell fluorescence, using the spectrofluorometer Wallac Victor2 multiwell instrument (Perkin-Elmer, Monza, Italy). The calibration curve was obtained by reporting the number of cells in each well on a graph with the relative spectrofluorometer reading.

### 2.5. Resveratrol

Standard trans-resveratrol was purchased from Sigma, Milan (Figure 1a). The trans-resveratrol diastereomeric mixture was obtained from the isomerization of trans-resveratrol in our laboratories as follows: 10 mg/mL of a standard solution of trans-resveratrol in acetonitrile/water 50:50 was isomerized by UV irradiation. UV irradiation was performed with a Helios Italquartz UV lamp at 366 nm for 3 h. This time lapse was needed to obtain a 50:50 trans/cis-resveratrol mixture. The isomerization process was controlled by capillary electrophoresis, as reported previously [36].

### 2.6. Determining Antioxidant Capacity

Antioxidant capacity was tested with the OxiSelect Hydrogen Peroxide Assay Kit Colorimetric (Cat. No STA-343, Cell Biolabs, Inc., San Diego, CA, USA) following the manufacturer’s instructions. The assay was carried out in triplicate in 96-well plates.

### 2.7. Cytotoxicity Assay

Cells were seeded in triplicate in 24-well plates and incubated for 24 h. Culture cells were exposed to 1.25 and 2.5 μM trans-resveratrol or diastereomeric mixture for 18 h. The cell monolayer was exposed to 250 μM CuOOH (Sigma-Aldrich) for 10 min. Two controls were included in these experiments. One control is represented by untreated cells, i.e., without resveratrol and CuOOH, whereas the other control is represented by cells treated with CuOOH, without resveratrol pre-treatment.

### 2.8. Cell Counting and Proliferation

Cell number was evaluated using the Wallac Victor2 spectrofluorometer by reading the fluorescence emitted from the attached cells. The number of cells was calculated using the calibration curve obtained with wells containing a known number of cells. Cell proliferation was quantitatively measured using the Alamar Blue Cell Viability Reagent (Invitrogen). Cell proliferation within samples was evaluated using the interpolation method, by comparing the percentage of Alamar Blue reduction in the sample to the standard curve [37,38].

### 2.9. Statistical Analysis

Statistical analyses were performed by Prism software. Results were obtained from four independent experiments and expressed as a mean value and SD. Student’s *t*-test was used to evaluate the statistical significance of the differences in mean values; *p* values < 0.05 were considered significant.

## 3. Results

### 3.1. Cell Clones

Parental human keratinocytes (NCTC-2544), transfected with the recombinant plasmid expressing the green fluorescent protein (pIRES2-AcGFP1), were cultured in the presence of 300 µg/mL of Geneticin. A total of 28 G418 resistant and fluorescent cell clones, i.e., NCTC/GFP, were isolated and expanded as independent cells. One out of these twenty-eight cell clones was expanded as a cell line and employed herein to perform all the experiments. This clone showed homogeneous cell characteristics. AcGFP expression was assessed by fluorescence microscopy observation, while its stability was confirmed over a period of 12 weeks as fluorescence intensity by spectrofluorimetric analysis (Figure 1b).

### 3.2. Cytological Characterization

**Figure 2:**
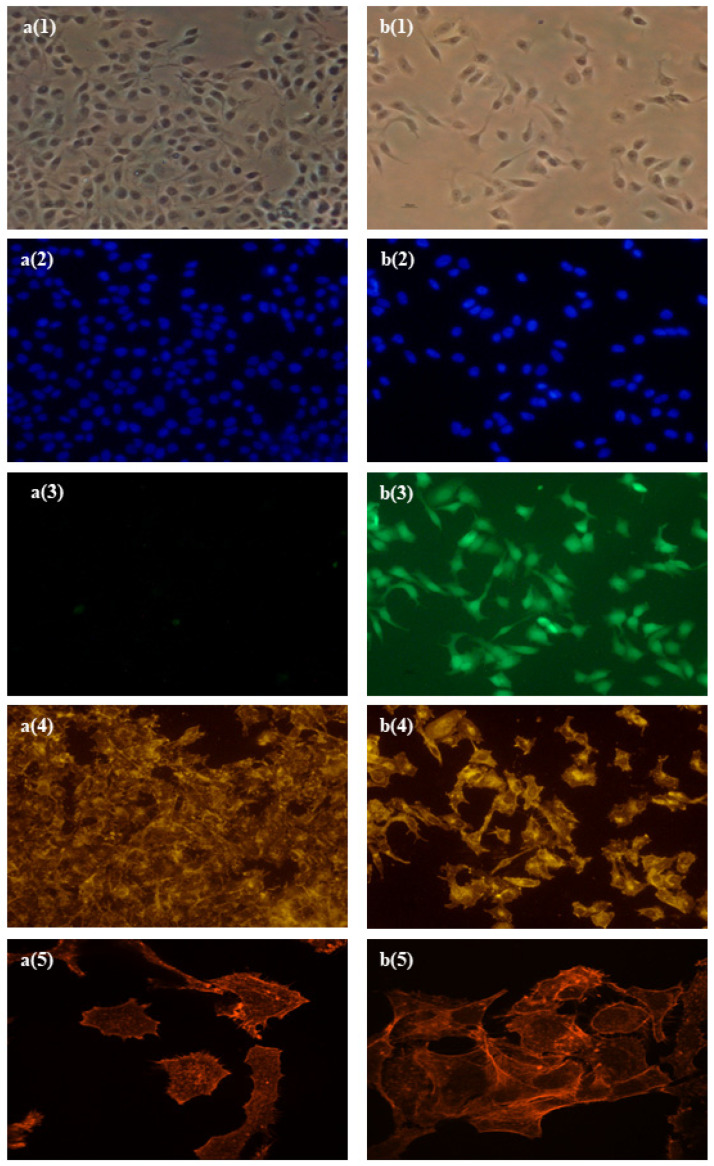
Microphotographs showing parental cells NCTC_2544 (**a**) and engineered cells NCTC_2544_AcGFP (**b**). Panels (**a**(**1**),**b**(**1**)) are microphotographs with white light to show cell morphology and shape; panels (**a**(**2**),**b**(**2**)) are microphotographs with nuclei counterstained with DAPI; panels (**a**(**3**),**b**(**3**)) are microphotographs with fluorescence light to visualize constitutive green fluorescence; panels (**a**(**4**),**b**(**4**)) are microphotographs with fluorescence light to visualize actin fiber architecture (magnitude 20×; panels (**a**(**5**),**b**(**5**)) are microphotographs with fluorescence light to visualize actin fiber architecture (magnitude 60×).

Comparative molecular characterization was carried out with the parental NCTC-2544 cell line and NCTC_2544_AcGFP engineered cells. Actin fibers were visualized using Phalloidin-TRIC conjugated specifically in order to assess whether the exogenous protein could alter NCTC_2544_AcGFP cytoskeletal organization. The cytoskeletal architecture of the parental and engineered cells was indistinguishable (Figure 2). Therefore, the presence of the exogenous protein AcGFP does not affect the cytoskeletal organization of the engineered cells.

### 3.3. Determining Antioxidant Capacity

**Figure 3:**
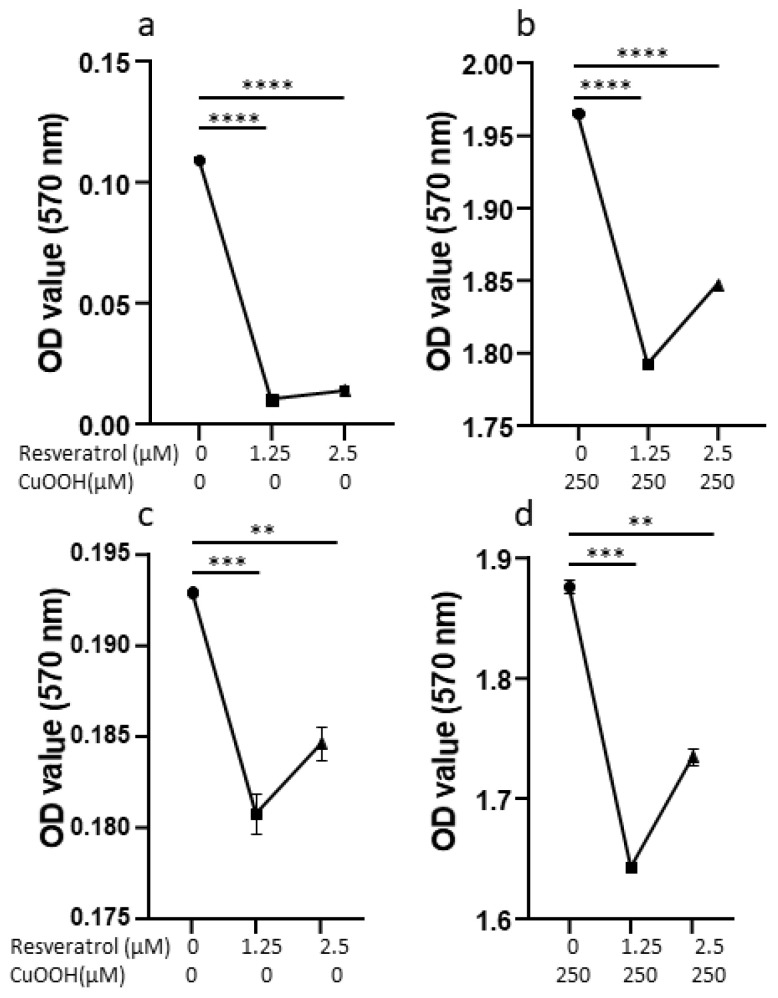
Effect of trans-resveratrol on oxidative stress on non-treated cells (panel (**a**)) and effect of trans-resveratrol on oxidative stress on cells treated with CuOOH (panel (**b**)). Horizontal bars indicate a statistically significant difference. Effect of resveratrol diastereomeric mixture on oxidative stress on non-treated cells (panel (**c**)) and effect of trans-resveratrol on oxidative stress on cells treated with CuOOH (panel (**d**)). Horizontal bars indicate statistically significant differences (**: *p* < 0.005; ***: *p* < 0.0005; ****: *p* < 0.00005).

The antioxidant capacity of resveratrol was tested using the OxiSelect Hydrogen Peroxide Assay Kit Colorimetric. Results were shown in Figure 3.

### 3.4. Cell Number Counting

Since NCTC/GFP cells express green fluorescent protein constitutively, fluorescence intensity may depend exclusively on the number of cells within a sample. To confirm the linearity of fluorescence intensity with the cell number, samples sets containing 3.125 × 10^3^, 6.25 × 10^3^, 1.25 × 10^4^, 2.5 × 10^4^, 5 × 10^4^, and 1 × 10^5^ cells/cm^2^ were analyzed by spectrofluorometer. The graphical expression of these experiments confirmed that fluorescence intensity values (absolute measure) are proportional to the number of cells within a sample. On this basis, having gained the fluorescence value, it was possible to extrapolate the number of cells contained within a sample by interpolation with the standard curve (Figure 1c).

### 3.5. Effect of CuOOH on the Human Keratinocyte NCTC/GFP Cell Line

Cells were treated with 250 μM CuOOH for 10 min. Spectrofluorimetric analysis showed that the oxidant agent caused a reduction in NCTC-GFP cells from 9.6 × 10^4^ to 6.1 × 10^4^ (*p* < 0.0005). Moreover, the Alamar Blue assay showed a proliferation reduction from 1.1 × 10^5^ to 9 × 10^4^ (*p* < 0.05). The two cell number reductions are statistically significant, as shown by the *p* values (Figure 1d).

### 3.6. Resveratrols

In general, cis-resveratrol is less pervasive in natural products than trans-resveratrol, thereby justifying a lack of analytical standards and difficulties in its identification and quantification. Notwithstanding this, the possibility of converting trans-resveratrol to cis-resveratrol by UV irradiation has previously been described in literature [39]. In a previous study we reported on the effect of irradiation time on the isomerization of trans-resveratrol to equilibrium conditions with an 80% final conversion [40].

**Figure 4:**
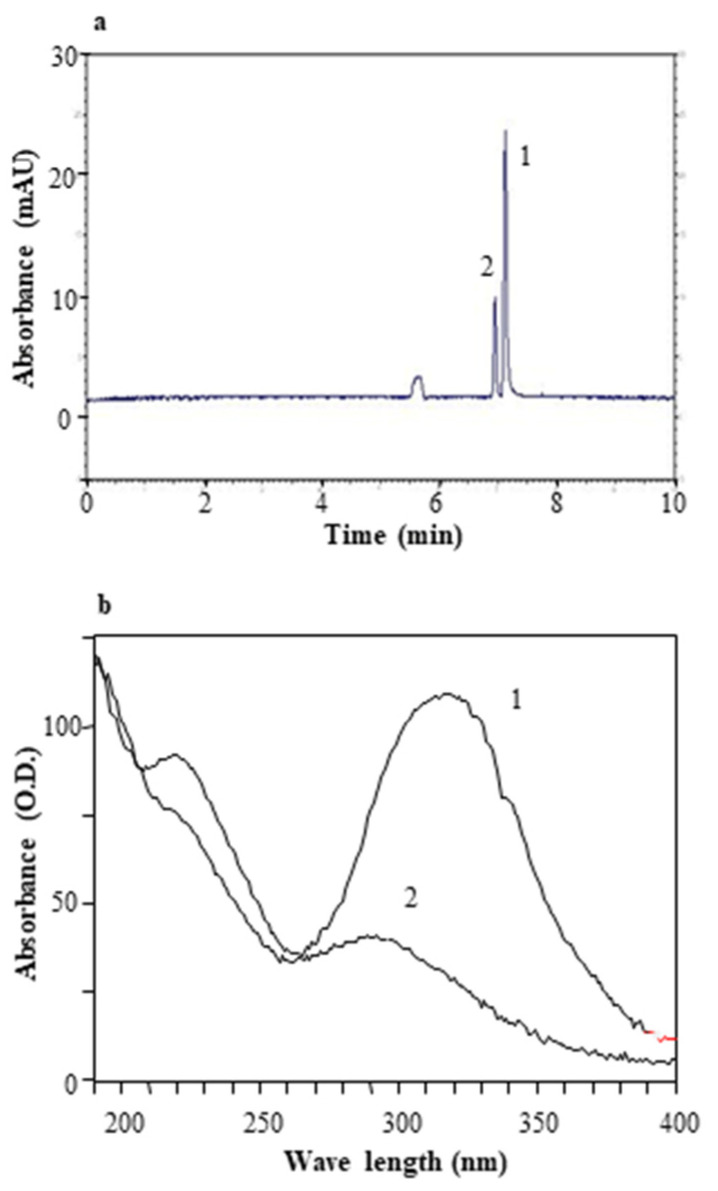
Electropherogram of trans-resveratrol standard solution and absorption spectrum. Panel (**a**): Electropherogram of trans-resveratrol standard solution diluted 1:100 after 90 min of isomerization (peak 1 = trans-resveratrol; peak 2 = and cis-resveratrol). Panel (**b**): Comparison between absorption spectrum of standard (trans-resveratrol (1) absorption spectrum and cis-resveratrol (2) absorption spectrum).

Every 30 min, a portion of isomerized trans-resveratrol standard solution was diluted 1:100 and injected in order for the analysis to verify the isomerization percentage. The electropherogram of isomerized trans-resveratrol standard solution, after 90 min, was reported in Figure 4a. At this time the mixture was made up of 30% cis-resveratrol and 70% of trans-resveratrol. Trans-resveratrol (1) and cis-resveratrol (2) peaks were identified by comparing retention times and their absorption spectrum. The absorption spectrum maximum reading for trans-resveratrol (1) is at 315 nm, while for cis-resveratrol (2) it is at 290 (Figure 4b). After 3 h, trans-resveratrol isomerization was at 50%. The resulting solution was lyophilized overnight in a Christ α 1–2 lyophilizer, and the obtained product was used for the biological assay.

### 3.7. Effect of Resveratrol on the Cell Numbers

Cells (*n* = 5 × 10^4^/cm^2^) were pre-incubated with resveratrol in our experimental conditions. Cell numbers increased both on CuOOH-treated and non-treated cells when evaluated using the spectrofluorometer.

**Figure 5:**
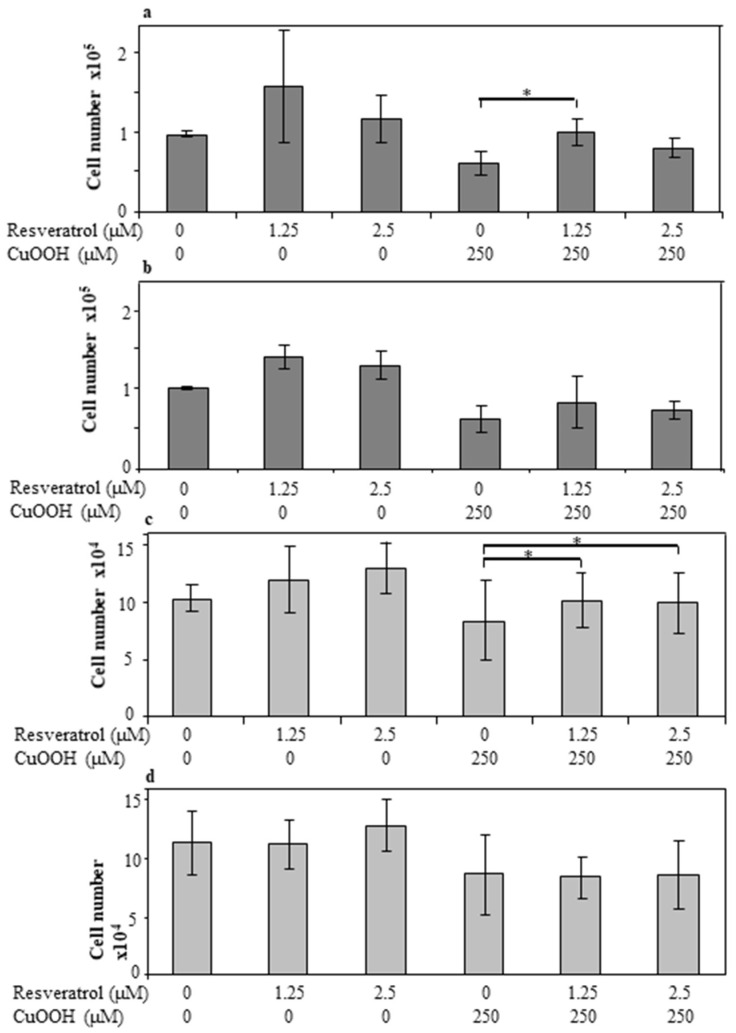
Effect of trans-resveratrol (panel (**a**)) and diastereomeric mixture (panel (**b**)) treatment on cell numbers evaluated by the measuring fluorescence. Horizontal bars indicate a statistically significant difference (*: *p* < 0.05). Effect of trans-resveratrol (panel (**c**)) and diastereomeric mixture (panel (**d**)) treatment on cell proliferation evaluated by Alamar Blue assay. Horizontal bars indicate a statistically significant difference (*: *p* < 0.05).

Specifically, cell numbers increased by a statistically significant difference when 1.25 μM of trans-resveratrol was used on CuOOH-treated cells, from 6 × 10^4^ to 1 × 10^5^ (*p* < 0.05). On the other hand, cell numbers still increased upon using trans-resveratrol at a concentration of 2.5 μM, but the difference was not statistically significant. The same result, in terms of non-statistically significant differences in the cell number, was obtained by pre-incubating CuOOH non-treated cells with trans-resveratrol at 1.25 μM and 2.5 μM. Similarly, both CuOOH-treated and non-treated cells, when pre-incubated with 1.25 μM and 2.5 μM diastereomeric mixture, showed no statistically significant increase in cell numbers (Figure 5a,b). Higher concentrations of the diastereomeric mixture and of trans-resveratrol caused a mild increase in the cell numbers or reduced human keratinocyte number, but the difference was not statistically significant (data not shown).

### 3.8. Effect of Resveratrol on Cell Proliferation

Proliferation increased in cells that had been pre-incubated with resveratrol, both in cells treated with CuOOH and in the control. Data were obtained using the Alamar Blue assay. The increase in cell proliferation in CuOOH-treated cells was statistically significant when two different concentrations of trans-resveratrol were employed, i.e., 1.25 μM and 2.5 μM. The increases were from 8.4 × 10^4^ to 1 × 10^5^ (*p* < 0.05) for 1.25 μM and from 8.4 × 10^4^ to 9.9 × 10^4^ (*p* < 0.05) for 2.5 μM, respectively. In the same experimental conditions, the resveratrol diastereomeric mixture caused a non-statistically significant increase in cell proliferation. The same result was obtained in CuOOH non-treated cells, which have been pre-incubated with both the trans-resveratrol and diastereomeric mixtures, at a concentration of 1.25 μM and 2.5 μM, respectively (Figure 5c,d). Cell proliferation data, obtained on cells pre-incubated with higher concentrations (5 μM) of the diastereomeric mixture and trans-resveratrol, showed a very low increase in cell proliferation or even a reduction (data not shown).

## 4. Discussion

In this comparative study the antioxidant effects of trans-resveratrol and its diastereomeric mixture were determined in fluorescent-engineered human keratinocytes. As reported previously, we herein show that the presence of AcGFP does not affect cytoskeletal organization [41], and that fluorescence intensity is proportional to the number of cells [41,42]. The high expression level of trans-gene in the genetically modified NCTC/GFP cell line ensured a direct proportion between fluorescence intensity and cell number [41,43].

In a recent study, human keratinocytes HaCaT, which had been irradiated with UV A to induce oxidative stress, were treated with increasing concentrations of trans-resveratrol. It was shown that both pre- and post-treatment with trans-resveratrol provided significant protection, as demonstrated by an increase in cellular activity and a decrease in ROS level resveratrol [44]. Another study conducted on HaCaT [45] showed that the toxicity of an elicitor was strongly attenuated by post-treatment with trans-resveratrol. Further studies focused their attention on the differences between the effects of the two resveratrol isomers in different experimental models. Indeed, it has been reported that the two isomers have similar levels of efficiency [46,47], while other investigations have found trans-resveratrol and cis-resveratrol to have different efficiency levels [48,49,50,51].

Our results indicate that keratinocytes incubated with CuOOH showed a reduction in cell number and proliferation index. Cells that had been pre-incubated with resveratrol were protected from damage. These data confirm and extend previous results on the effect of resveratrol on human epidermal keratinocytes [44,52,53]. Our comparative study with resveratrol isomers allowed for some differences in the behavior of trans-resveratrol and its diastereomeric mixture to be shown. The addition of trans-resveratrol to keratinocytes, protected the cells by inactivating the cytotoxic effects of CuOOH. Trans-resveratrol at a concentration of 1.25 μM caused a statistically significant decrease in oxidative stress and an increase in both cell number and proliferation. However, it should be noted that only the concentration of 2.5 μM showed a statistical significance in cell proliferation. The addition of trans-resveratrol to the control cells demonstrated an increase in cell number and proliferation at all concentrations tested, although without statistically significant differences. The diastereomeric mixture illustrated a protective effect against oxidative damage similar to that of trans-resveratrol, but with less efficacy. When the diastereomeric mixture was used on cells that had previously been treated with CuOOH, a non-statistically significant increase in both cell number and proliferation was obtained. Similar results were detected with the pre-incubation of CuOOH non-treated cells using the diastereomeric mixture, at all concentrations tested. Herein, the trans-resveratrol and diastereomeric mixtures, at concentrations of 1.25 μM and 2.5 μM, were used as reported in previous studies [44,54,55,56]. When higher concentration (5 μM) of both the trans-resveratrol and diastereomeric mixture was employed, a less protective effect was obtained, whereas in some cases, a negligible depletion in cell number and inhibition of proliferation was observed. These data are in agreement with the results reported by Holian et al. [43]. In our experimental model, CuOOH and resveratrol were used as a free-radical promoter and antioxidant molecule, respectively. CuOOH, a lipophilic compound, may act in the phospholipidic bilayer structure of the cell membrane where it induces lipid peroxidation and generates ROS, which may diffuse from the membrane lipid bilayer into the intracellular compartment [35]. Resveratrol has been reported as contrasting oxidative stress by acting as a radical scavenger [45,57].

Resveratrol pre-incubation of cells may eliminate, at least in part, the ROS generated by CuOOH. As a consequence, adding resveratrol led to a protective effect on the keratinocytes. In addition, previous studies demonstrated (i) the presence of resveratrol binding sites in human keratinocytes [56] and (ii) that trans-resveratrol protects these cells from oxidative stress [53]. The protective effects of resveratrol against the oxidative agent are mediated by its ability to enhance the cellular production of antioxidant enzymes, such as glutathione S-transferase (GST), glutathione peroxidase (Gpx), NAD(P)H, quinone oxidoreductase 1 (NQO1), catalase (cat), and superoxide dismutase (SOD). Resveratrol activity is mediated along the ERK pathway, which allows the transcription of genes encoding antioxidant enzymes. Resveratrol also modulates multiple signaling mechanisms, while displaying an effect on the NF-κB and sirtuin/FoxO3a pathways [58].

## 5. Conclusions

Herein, the innovative cellular model represented by fluorescent-engineered keratinocyte cells allowed us to confirm that resveratrol has the ability to protect human cells from oxidative stress. Specifically, trans-resveratrol proved to be more effective than the diastereomeric mixture. This characteristic could be ascribed to the spatial position of the hydroxyl groups that are of prime importance in terms of its chelating capacity [10]. Similarly, how far resveratrol activity protects cells from oxidative stress, depends on the hydroxyl group position. Our data indicate that fluorescent-engineered keratinocytes may represent a rapid, low-cost cellular model for measuring the antioxidant effects of trans-resveratrol and diastereomeric mixture indirectly.

## Data Availability

All of the data are contained within the article.
